# Extraction of Unerupted Maxillary Canine Teeth in a Maned Wolf (*Chrysocyon brachyurus*)

**DOI:** 10.1155/2016/2827647

**Published:** 2016-07-11

**Authors:** Lourdes M. B. Pessoa, Marcello Roza, Anderson Farias, Pedro Henrique de Jesus, Rita de Cassia Campbell, Mariângela Pereira de Pinho

**Affiliations:** ^1^Parque Fioravante Galvani/Instituto Lina Galvani, Rodovia BR-242, Km 870, Zona Rural, 47800-000 Barreiras, BA, Brazil; ^2^Division of Veterinary Medicine, Federal University of Goias, Avenida Esperança, s/n, Setor Itatiaia, 74690-900 Goiânia, GO, Brazil; ^3^OdontoZoo Veterinary Dentistry, Quadra 42 Conjunto A, Casa 02, Gama Centro, 72465-420 Brasilia, DF, Brazil; ^4^Faculty of Veterinary Medicine, Pioneer Union of Social Integration, Fazenda Lagoa Bonita, 70390-125 Planaltina, DF, Brazil

## Abstract

The purpose of this case report is to describe the diagnosis and treatment of unerupted canine teeth in a maned wolf. After physical examination, complete blood count, and serum biochemical profile, the animal underwent general anesthesia and head radiography was performed to confirm the diagnosis. The treatment consisted of the extraction of both maxillary canine teeth and clinical and radiographic follow-up of the right mandibular canine tooth.

## 1. Introduction

The maned wolf (*Chrysocyon brachyurus*) is a characteristic canid of the cerrado biome and its existence area includes Central Brazil, Paraguay, and small areas of Argentina, Bolivia, Peru, and possibly Uruguay. Although the conservation of this species is fundamental, mainly to the equilibrium of the cerrado biome, the maned wolf has been listed as “near threatened” on the IUCN Red List [1].

Unerupted teeth are those that appear to be missing when we examine the animal's mouth but are in fact present either beneath the surface of the bone of the jaw or under the gum [2]. A study involving 627 dogs reported only six impacted teeth [3].

Unerupted teeth may be due to obstruction from crowding or from some other physical barrier. They may occasionally be due to an abnormal eruption path, presumably because of unusual orientation of the tooth germ [4].

The purpose of this case report is to describe the diagnosis and treatment of unerupted canine teeth in a maned wolf.

## 2. Case Presentation

**Figure 1:**
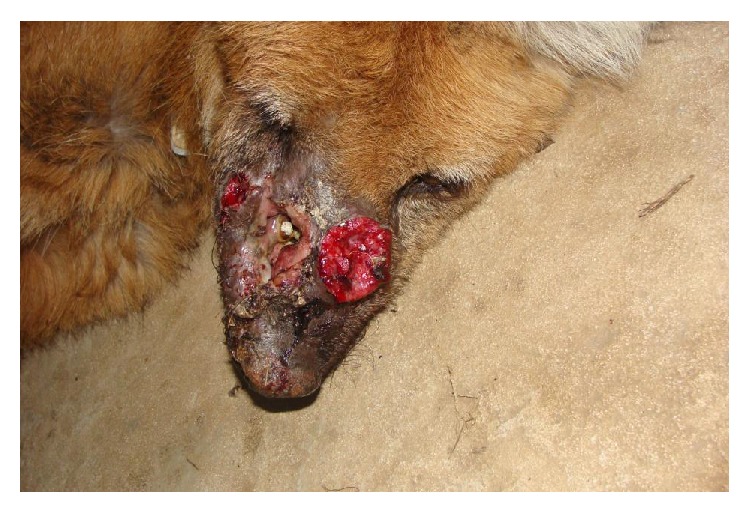
Dorsoventral view of the head of the maned wolf with infraorbital swellings, abscess formation, and fistulation.

A maned, male, juvenile wolf, weighing 25 kg, was rescued and brought to the Fioravante Galvani Park, in Bahia, Brazil, two years ago. The animal had difficulty in eating solid foods and presented bilateral infraorbital swellings, resulting in abscess formation and fistulation (Figure 1).

**Figure 2:**
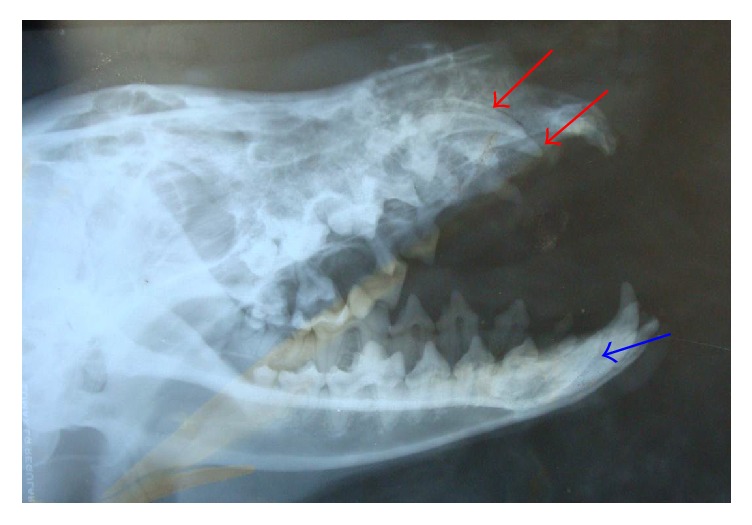
Laterolateral (oblique) head radiography of a maned wolf showing two unerupted maxillary canine teeth (the red arrow) and an unerupted right mandibular canine tooth (the blue arrow).

An oral examination revealed the absence of both maxillary canine teeth and the right mandibular canine tooth. A head radiography revealed two unerupted maxillary canine teeth and an unerupted right mandibular canine tooth (Figure 2). These teeth had open apices and thin dentinal walls.

The animal was submitted to preoperative examinations (total protein, albumin, ALT, ALP, creatinine, and complete blood count) and food was withheld for 12 hours prior to anesthesia. The anesthetic protocol was as follows: sedation and analgesia, ketamine (8 mg/kg, IM), midazolam (0.3 mg/kg, IM), and meperidine (5 mg/kg, IM); induction, propofol (5 mg/kg, IV); maintenance, inalatory isoflurane and oxygen. Lactated Ringer's solution (10 mL/kg IV) was administered throughout anesthesia [5]. Antibiotic and anti-inflammatory therapy was administered preoperatively and for five days postoperatively.

**Figure 3:**
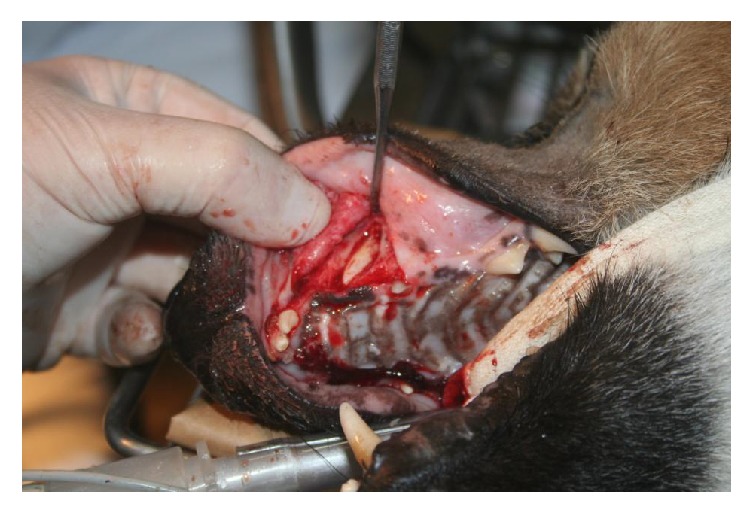
Labial alveolectomy during open extraction of the left maxillary canine tooth.

Based on the clinical and radiological findings, the maxillary canine teeth were extracted. Labial mucoperiosteal flap was raised to expose the bone overlying the unerupted teeth. A burr on a slow-speed handpiece under irrigation with sterile saline was used to perform alveolectomy (Figure 3). A dental elevator and extraction forceps were used to complete the extraction. After complete extraction, the remaining alveolus was debrided with a curette and irrigated with sterile saline solution. The flap was sutured with 4-0 polyglyconate in a simple, interrupted pattern. There were no postoperative complications, and the maned wolf recovered well.

## 3. Discussion

The authors found no studies or reports about unerupted teeth in maned wolves. A study involving eighty specimens of maned wolf (63 skulls and 17 living animals) suggested that the most common oral diseases include tooth wear (83.7%) and tooth fracture (54.4%) [6], but unerupted teeth were not reported. The same study revealed that periodontal disease is common in maned wolf [7].

Injuries or systemic diseases occurring during dental development can have an impact on tooth eruption and dental occlusion [6, 8]. The maned wolf in the present case report was rescued as a juvenile after an accident without obvious injuries. Subsequently, it developed infraorbital swellings with abscess formation and fistulation. After a more thorough examination unerupted maxillary canine teeth were diagnosed. Their unerupted state could have been caused by previous trauma as suggested elsewhere [6, 8, 9].

The results of this report show that we can use the diagnostic and therapeutic approach in maned wolf just like those used in dogs and provide marked improvement in the quality of life of the animal. Further studies are necessary to elucidate the distribution of oral diseases in wild canids.
